# Supplementary material for: Development of a computer-aided design software for the quantitative evaluation of aesthetic damage
Source: PLoS One. 2019 Dec 18;14(12):e0226322. doi: 10.1371/journal.pone.0226322 (PMC6919621; doi:10.1371/journal.pone.0226322)
Supplement: S1 Code site — 1. (ZIP) [file pone.0226322.s001.zip › [EESC_jr][Projeto]Relatorio_07_21/reports/templates_zip/formwizard-master.zip!/formwizard-master/examples/example_17_cancel_the_submit_in_using_beforeSubmit_beforeSend_and_beforeSerialize_callbacks.html]

xml version="1.0" encoding="UTF-8"?


JQuery Form Wizard


### Example of how to cancel the post using the beforeSubmit, beforeSend or beforeSerialize callbacks

- Straight wizard with three steps.
- Validation plugin enabled for the email field on the second step.
- Form plugin enabled and beforeSerialize, beforeSubmit, beforeSend and success callbacks are used.
- It is possible to cancel the post at each of the before\* callbacks

---


First step - Name  
First name  
  
Surname

Step 2 - Personal information  
Social Security Number  


 -
  
Phone number  
 -
 -
  
\*Email

Last step - Username  
User name  
  
Password  
  
Retype password


---
